# ALK gene copy number gains in non-small-cell lung cancer: prognostic impact and clinico-pathological correlations

**DOI:** 10.1186/s12931-016-0422-8

**Published:** 2016-08-25

**Authors:** U. Peretti, R. Ferrara, S. Pilotto, S. Kinspergher, M. Caccese, A. Santo, M. Brunelli, A. Caliò, L. Carbognin, I. Sperduti, M. Garassino, M. Chilosi, A. Scarpa, G. Tortora, E. Bria

**Affiliations:** 1 Medical Oncology, University of Verona, Azienda Ospedaliera Universitaria Integrata, P.le L.A. Scuro 10, 37124 Verona, Italy; 2 Department of Pathology and Diagnostics, University of Verona, Azienda Ospedaliera Universitaria Integrata, P.le L.A. Scuro 10, 37124 Verona, Italy; 3 Biostatistics, Regina Elena National Cancer Institute, Via Elio Chianesi 53, 00144 Rome, Italy; 4 Istituto Nazionale Tumori, Milan, Italy; 5 ARC-NET Applied Research on Cancer Center, University of Verona, P.le L.A. Scuro 10, 37124 Verona, Italy

**Keywords:** Lung cancer, Anaplastic lymphoma kinase, Copy number gain, Prognosis, Clinico-pathological characteristics

## Abstract

**Background:**

The correlation between ALK gene copy number gain (ALK-CNG) and prognosis in the context of advanced non-small-cell lung cancer (NSCLC) remains a controversial issue. This study aimed to evaluate the association among ALK-CNG according to Fluorescent In Situ Hybridization (FISH), clinical characteristics and survival in resectable and advanced NSCLC.

**Methods:**

Clinical and pathological data of patients with resectable and advanced NSCLC were retrospectively collected. Tumor tissues were analyzed for ALK-CNG by FISH, and patients were divided in 3 groups/patterns on the basis of ALK signals: disomic [Pattern A], 3–7 signals [Pattern B], >7 signals [Pattern C]. The association between clinical and pathological features and ALK-CNG patterns was evaluated. Disease/progression-free and overall survival (DFS/PFS and OS) were estimated using the Kaplan-Meyer method.

**Results:**

A number of 128 (76.6 %) out of the 167 eligible patients were evaluable for ALK-CNG, displaying pattern A, B and C in 71 (42.5 %), 42 (25.1 %) and 15 (9 %) patients, respectively. Gains in ALK-CNG appear to be more frequent in smokers/former smokers than in non-smokers (74.2 % versus 20.4 %, respectively, *p* = 0.03). Pattern A and C seem more frequently associated with higher T-stage (T3-4), while pattern B appears more represented in lower T-stage (T 1-2) (*p* = 0.06). No significant differences in survival rate were observed among the above groups.

**Conclusions:**

A high ALK-CNG pattern might be associated with smoking status and theoretically it might mirror genomic instability. The implications for prognosis should be prospectively investigated and validated in larger patients’ series.

**Trial registration:**

We confirm that all the study was performed in accordance with relevant guidelines and regulations and that all the protocol (part of a larger project MFAG 2013 N.14282) was approved by the local Ethics Committee of the Azienda Ospedaliera Universitaria Integrata of Verona on November 11st, 2014.

## Background

Nowadays, non-small-cell lung cancer (NSCLC) might be considered as a universe of different diseases. Particularly in the context of adenocarcinoma, reliable evidence is available suggesting that cancer development and progression might be led by the addiction from aberrant pathways triggered by genetic abnormalities acting as oncogenic *drivers* (such as the activating mutation of EGFR or the translocation of ALK). In this setting, the inhibition of these *drivers* with selective agents has radically changed the natural history of the disease [1–7]. Unfortunately, only a limited subpopulation of lung cancer patients might benefit from this personalized treatment. Hence, the importance of identifying and validating new molecular alterations with prognostic and predictive significance in order to extend the proportion of lung cancer patients who might benefit from targeted drugs.

ALK is a versatile oncogene whose role has been recognized in a large variety of tumors through different activation mechanisms, mainly the chromosomal rearrangement with different fusion partners (as the microtubule associated protein EML4 in NSCLC or the nucleophosmin NPM1 in anaplastic large cell lymphoma). In NSCLC, the successful history of ALK inhibitors started with crizotinib and is still ongoing [8]. Crizotinib is an orally available tyrosine kinase inhibitor (TKI) originally designed to target the mesenchymal to epithelial transition process, but it also potently inhibits ALK and ROS1 phosphorylation and signaling [9]. Based on encouraging preclinical data, impressive preliminary results were published from an expansion cohort of a phase I trial [10] and from the PROFILE 1005 phase II trial [11]. In the second-line trial PROFILE 1007, 347 patients with ALK-rearranged NSCLC pretreated with a platinum doublet received either crizotinib or second-line chemotherapy with docetaxel or pemetrexed. Patients benefited from crizotinib in both terms of overall response rate (ORR) (65 % versus 20 %; *p* < 0.001) and median progression-free survival (PFS) (7.7 versus 3.0 months; HR 0.49; *p* < 0.001) [6]. Moreover, in the first-line phase III trial PROFILE 1014, 343 treatment-naive patients with ALK-rearranged NSCLC were randomized to receive either crizotinib or standard platinum-based plus pemetrexed first-line chemotherapy. Also in this setting, crizotinib met not only the primary end point, achieving a significantly longer median PFS (10.9 versus 7.0 months; HR 0.45; *p* < 0.001), but also a significantly higher ORR (74 % versus 45 %; *p* < 0.001). The more frequent adverse events associated with the administration of crizotinib were vision disorders, diarrhea, edema, increased aminotransferase levels and neutropenia [7]. The main limitations of the crizotinib efficacy are represented by its low brain penetrance and its inactivity against secondary mutations of the ALK gene. Therefore, clinical trials evaluating next generation ALK inhibitors are currently ongoing. Promising response rates and PFS have been reported particularly in crizotinib-refractory ALK-rearranged NSCLC patients treated with ceritinib [12–14], alectinib [15, 16], brigatinib [17] and lorlatinib [18]. Recently, the pre-planned interim analysis of the J-ALEX trial, demonstrated the superiority of alectinib to crizotinib in untreated ALK-rearranged NSCLC patients (median PFS not reached versus 10.2 months; HR 0.34; *p* < 0.0001) [19].

Another ALK aberration, the gene copy number gain (CNG), has been identified in several tumor types and significantly correlated with poor prognosis and/or advanced disease status.

In this regard, ALK-CNG has been reported in the 10 % of renal cell carcinoma patients and the presence of more than 5 copies of the ALK gene was significantly associated with high tumor size, nuclear grade and worse 10-years survival rate [20]. In colorectal cancer patients, ALK-CNG was found in the 3.4 % of non-molecularly selected patients and in the 37 % of RAS-BRAF-PI3KCA wild-type patients [21, 22]. In both studies, the ALK gene copy number increase was significantly associated with poor prognosis. Moreover, in *triple wild-type* patients, the response rate to cetuximab or panitumumab was significantly higher in the subgroup of disomic ALK (70 %) as compared with ALK-CNG subgroup (32 %), with similar results in term of PFS and overall survival (OS), raising the hypothesis of a possible role of ALK-CNG in resistance to anti-EGFR therapy [21, 22]. ALK-CNG was further identified in the 13 % of patients affected by hepatocellular carcinoma, negative for serum hepatitis B virus DNA, with a significant correlation among ALK-CNG (≥4 copies versus < 4 copies), 3-year PFS rate (27 % versus 42 %) and 3-year OS rate (18 % versus 47 %) [23]. In rhabdomyosarcoma (RMS), ALK-CNG was detected in the 88 % of alveolar RMS and in the 52 % of embryonal RMS (ERMS). In ERMS, specific ALK gain in the primary tumor correlated with metastatic disease and poor 5-year disease-specific OS (62 versus 82 %) [24]. Moreover, ALK-CNG was retrospectively detected in the 47.2 % of patients with inflammatory breast cancer and significantly correlated with worse overall survival (24.9 versus 38.1 months) and recurrence free survival (RFS) after curative mastectomy (12.7 versus 43.3 months) compared to ALK-CNG negative patients [25]. Finally, an aberrant activation of ALK, both by activating mutation and amplification, might drive the tumorigenesis of neuroblastoma (NBL) [26]. In a retrospective analysis of pediatric patients with NBL, ALK amplification due to polyploidy (ALK/CEP2 ratio > 4) represented a negative prognostic factor for OS [27].

Regarding lung cancer, the presence and potential prognostic role of the different ALK gene aberrations (translocation and gene copy number gains) have been widely investigated with debatable results (as more extensively revised in the discussion paragraph). Our study, retrospectively conducted in a large series of NSCLC patients, aimed to investigate the potential correlation between ALK-CNG and clinical features, exploring in particular the prognostic implications of this genetic abnormality in resected and advanced NSCLC patients.

## Methods

### Patients and samples

All the reported data of this study were obtained from the Lung Verona Database (Verona, Italy). Clinical (gender, familiarity, comorbidity, performance status, smoking history, treatment) and pathological (TNM, disease stage, grading) information has been collected. All the cases were classified according to WHO criteria [28]. Appropriate samples, containing at least 90 % neoplastic cells, have been selected for the interphase cytogenetic and molecular studies.

### Interphase fluorescence in situ hybridization (FISH) analysis

#### ALK gene status assessment by break-apart probe

Interphase cytogenetic analysis was performed by FISH using 5 μm sections from formalin-fixed and paraffin-embedded tissues and a commercially available break-apart ALK kit (Abbott-Vysis) that uses two DNA probes on the ALK gene, one at the 3’ and one at the 5’ regions. The slides were examined using an Olympus BX61 (Germany) with appropriate filters for SpectrumOrange, SpectrumGreen and the UV filter for the DAPI nuclear counterstain. The signals were recorded with a CCD camera (CytoVysion, Olympus) and digitalized by Fluo/D-SIGHT (Menarini/Visia Imaging). A total of 150 neoplastic nuclei were assessed in at least three different areas for surgical specimens. In bioptic samples all neoplastic nuclei (on average 60 nuclei) were evaluated. Nuclei harboring split-signals were scored as positive for ALK rearrangement using the approved cut-off (15 %). Gene copy number was initially scored per each case.

#### Control probes using centromeric alpha-satellite specific for chromosome 2, 3 and 17 (CEP2, CEP3 and CEP17) probes

Centromeric alpha-satellite specific for chromosome 2, 3 and 17 (CEP2, CEP3 and CEP17) were used as control probes (Vysis-Abbott, Olympus, Rome, Italy). Control probes polysomy was detected by performing FISH assay on adjacent serial tissue sections. Briefly, each probe was diluted 1:10 in tDenHyb2 buffer (Insitus, Albuquerque, NM). Ten microliters of diluted probe were applied to each slide and cover slips were placed over the slides. Denaturation was achieved by incubating the slides at 80 °C for 10 min in a humidified box; then hybridization was done at 37 °C for 16 h. The cover slips were then removed and the slides were immersed at room temperature in 0.5 x SSC (Superconducting Super Collider) for 2 min and in 2 x SSC for 2 min. The slides were air dried and counterstained with 10 μl DAPI/Antifade (DAPI in Fluorguard, 0.5 μg/ml, Insitus, Albuquerque, NM). Fluorescent in situ signals were evaluated on carcinomatous and normal pulmonary adjacent parenchyma. A total of 150 neoplastic nuclei were assessed in at least three different areas for surgical specimens while 60 neoplastic nuclei were evaluated in bioptic samples.

### ALK interpretation

Mean copy number for the ALK locus (LSI) and the centromeric (CEPs) probes CEP2, CEP3 and CEP17 were primarily evaluated. A mean copy number of centromeric probes were secondly detected to assess ploidy. Ratio between mean copy number of ALK gene and mean copy number of control centromeric probes CEP2, CEP3 and CEP17 was finally scored. Amplification of the ALK locus gene was interpreted when the ratio (LSI/CEPs) was ≥ 2. When increasing gene copy number resulted < 2 after corrections by control probes (LSI/CEPs) the case was interpreted as having gains of chromosome due to polyploidy. Cases were digitalized by using the scan D-Sight/Fluo instrument (VisiaImaging, Florence, Italy).

### Statistical analysis

According to what stated by the Cytogenetics Laboratory of Verona’s University about the frequency distribution of ALK expression as a cytogenetic profile [29], patients were divided in three groups on the base of gene copy number (Pattern A: CNG = 2 [disomic]; Pattern B: CNG 3-7; Pattern C: CNG >7). The Fisher’s Exact Test (with a significance α error of 0.5) was applied to evaluate the correlation of ALK-CNG subgroups with the clinico-pathologic variables in the overall population and the treatment outcome in metastatic patients. In order to correct possible biases due to the disease stage frequencies, patients were divided in resected (those who underwent surgery for an operable disease) and metastatic patients (metastatic from the diagnosis). The prognostic analysis has been performed separately for these two groups. The hazard ratio (HR) and the 95 % confidence intervals (95 % CI) were estimated for each variable using the Cox univariate model. The OS (2-year survival rate) was calculated using the Kaplan Meyer’s method to identify a possible correlation between clinical outcome and expression of ALK. We estimated the disease free survival (DFS) for resected patients and the PFS for patients who underwent systemic treatment for metastatic disease. Finally, Kaplan Meyer curves were compared through Long Rank test and all the analyses were conducted using SPSS 18.0 software.

## Results

### Patients’ characteristics

A consecutive series of 205 NSCLC (112 biopsies, 93 surgical specimens) were collected from the Lung Verona Database. Thirty-eight patients with locally advanced or metastatic NSCLC harboring EGFR activating mutations and ALK rearranged tumors were excluded from the analysis. We analyzed a total of 167 patients and their clinico-pathological characteristics are summarized in Table 1. One hundred and six men (63.5 %) and 61 women (36.5 %) with a median age of 66 years (ranging from 29 to 85) were included. Among them, 56 patients (33.5 %) were smokers, 68 former smokers (40.7 %), 34 never smokers (20.4 %), while 9 patients (5.4 %) were not evaluable for smoking status; the 60.5 % of patients had familiarity for cancer; 25.7 % had cardiac and respiratory comorbidities. One hundred and one patients (60.4 %) were metastatic at the diagnosis; 26 patients (15.5 %) were stage I, 10 (6.1 %) stage II and 30 (18.0 %) stage III. The predominant histological subtype was adenocarcinoma with 147 cases (88 %), followed by squamous cell carcinoma with 7 cases (4.2 %) and mixed or other histotype were 11 cases (6.6 %). Among those patients evaluable for histologic tumor grade (69/167), 25.1 % were poor differentiated (G3). The majority of patients were in good clinical conditions with PS 0 (59.3 %) and 1 (21.6 %), while only the 7.1 % of patients were PS 2 or PS 3. Forty seven patients (28.1 %) affected by localized disease underwent surgery, in some cases followed by adjuvant chemotherapy; 3 patients were treated with neoadjuvant chemotherapy before surgery. Locally advanced patients were treated with chemo-radiotherapy in 17 cases (10.2 %). Seventy-five patients (44.9 %) with advanced disease were treated with chemotherapy tailored on the basis of histologic type, age, comorbidities and performance status. Overall, 89 patients with advanced disease were treated with I line chemotherapy, among them 3 patients (1.8 %) had complete response (CR), 31 (18.6 %) had partial response (PR), 20 (12 %) had stable disease (SD) and 35 patients (21 %) progressive disease (PD) (Table 1).

**Table 1:** Patients’ characteristics (167 evaluable patients for the clinical analysis)

	Patients number (%)
Gender	
Male	106 (63.5)
Female	61 (36.5)
Cancer familiarity	
Yes	101 (60.5)
No	43 (25.7)
Unknown	23 (13.8)
Comorbidity	
Yes	52 (31.1)
No	115 (68.9)
Performance Status sec. ECOG	
0	99 (59.3)
1	36 (21.6)
2–3	12 (7.1)
Unknown	20 (12.0)
Histology	
Adenocarcinoma	147 (88.0)
Squamous	7 (4.2)
Other/Mixed	13 (7.8)
T descriptor according to TNM	
1–2	102 (61.1)
3–4	65 (39.9)
N descriptor according to TNM	
0	36 (21.6)
1	19 (11.4)
2	66 (39.5)
3	46 (27.5)
M descriptor according to TNM	
0	66 (39.5)
1	101 (60.5)
Disease stage	
I-II	36 (21.6)
III-IV	131 (78.4)
Grading	
1	12 (7.2)
2	15 (9.0)
3	42 (25.1)
Unknown	98 (58.7)
Smoking status	
Current	56 (33.5)
Former	68 (40.7)
Never	34 (20.4)
Unknown	9 (5.4)
Starting treatment	
Surgery	47 (28.1)
Neoadjuvant	3 (1.8)
Chemo-radiotherapy	17 (10.2)
Chemotherapy	75 (44.9)
Support therapy	25 (15.0)
Response rate to 1^st^ line therapy	
CR	3 (1.8)
PR	31 (18.6)
SD	20 (12.0)
PD	35 (21.0)
NP	78 (46.6)
ALK-CNG	
disomic	71 (42.5)
3–7 copies	42 (25.1)
>7 copies	15 (9.0)
NA	39 (23.4)

*CR* complete response, *PR* partial response, *SD* stable disease, *PD* progressive disease, *NP* not performed, *CNG* copy number gain, *NA* not available

### Analysis of ALK gene copy number status

**Fig. 1:**
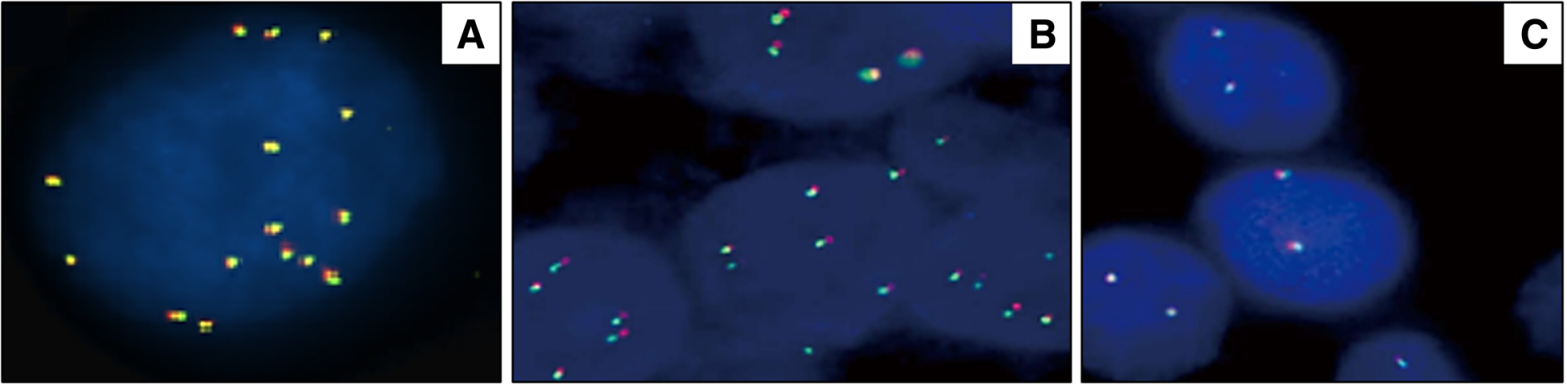
FISH findings in cell neoplastic nuclei. **a** Nucleus with >7 ALK fluorescent signals; **b** Nuclei with polysomy of ALK (from 3 to 7 ALK-CNG signals); **c** Nuclei with a disomic pattern of ALK

In the overall cohort of patients, 128 were available for ALK-CNG analysis. Fifty-seven cases (34.1 %; 95 % CI 34.2-47.7) showed ALK-CNG. We observed three clustered patterns of fluorescent signals (Fig. 1), applying the technique employed by our group for the study of the 3q chromosomal amplification in squamous cell lung carcinoma [29]. Fifteen patients (9.0 %) showed pattern C (>7 ALK fluorescent signals, ranging from 7 to 12) (Fig. 1-a); 42 patients (25.1 %) showed pattern B (from 3 to 7 ALK-CNG signals) (Fig. 1-b) and, as expected, the largest part of the samples (71 patients, 42.5 %) had pattern A (disomic) (Fig. 1-c) (Table 1). The three ALK patterns were interpreted using the control CEP2, CEP3 and CEP17 probes. Among pattern C, the 13 % (2 cases) showed a ratio ≥ 2 when corrected by CEP3 and CEP17. In the remaining 13 out of 15 cases with pattern C and in all the cases with pattern B the FISH results mirrored the mean number of centromeric signals with a final ratio < 2, classifiable as polyploidy. The only 2 cases showing ALK gene amplification were visible at fluorescent microscope as double minutes rather than homogeneously staining regions pattern.

### Association analysis between clinico-pathological characteristics of patients and ALK-CNG

No statistically significant correlations were observed between the three ALK-CNG patterns and the majority of the clinico-pathological characteristics (such as gender, histology, grading, comorbidity, cancer familiarity, performance status sec. ECOG) (Table 2). Nevertheless, the association analysis demonstrated that ALK-CNG, pattern B in particular, is more frequently detected in smokers and former smokers compared to never smoker patients (*p* = 0.03). Similarly, ALK-CNG seems to correlate with the primary tumor extension (T descriptor). Small tumors (T1 and T2) were more frequently detected in association with ALK-CNG pattern B compared to T3 and T4 tumors (43.1 % versus 22.8 %). No significant association has been observed between ALK-CNG and node (N descriptor) or metastatic (M descriptor) status. Although no statistically significant association has been reported between ALK-CNG and disease stage, early stages (I and II) are more represented in ALK pattern B, whereas in pattern C advanced diseases are more frequently detected (1 case stage I versus 14 cases stage III-IV). No well differentiated diseases (G1) have been observed in ALK pattern C subgroup (versus 8 cases G2-3). No difference in term of response to first line chemotherapy has been observed according to the different ALK-CNG patterns.

**Table 2:** Association analysis between clinico-pathological characteristics and ALK-CNG

Clinico-pathological parameters	ALK-CNG - patients number (%)				*p*-value
	Disomic	3–7	>7	Total	
Gender				128	0.59
Male	41 (51.9)	28 (35.4)	10 (12.7)	79 (100.0)	
Female	30 (61.2)	14 (28.6)	5 (10.2)	49 (100.0)	
Cancer familiarity				110	0.44
No	18 (54.5)	13 (39.4)	2 (6.1)	33 (100.0)	
Yes	41 (53.2)	25 (32.5)	11 (14.3)	77 (100.0)	
Smoking status				120	0.03
Never	20 (80.0)	4 (16.0)	1 (4.0)	25 (100.0)	
Current	24 (54.5)	13 (29.5)	7 (15.9)	44 (100.0)	
Former	22 (43.1)	23 (45.1)	6 (11.8)	51 (100.0)	
Comorbidity				121	0.19
No	23 (67.6)	8 (23.5)	3 (8.8)	34 (100.0)	
Yes	43 (49.4)	33 (37.9)	11 (12.6)	87 (100.0)	
Performance Status sec. ECOG				112	0.63
0	43 (57.3)	24 (32)	8 (10.7)	75 (100.0)	
1	12 (42.9)	11 (39.3)	5 (17.9)	28 (100.0)	
2–3	6 (66.7)	2 (22.2)	1 (11.1)	9 (100.0)	
Histology				128	0.83
Adenocarcinoma	63 (55.8)	38 (33.6)	12 (10.6)	113 (100.0)	
Squamous	3 (50.0)	2 (33.3)	1 (16.7)	6 (100.0)	
Other	5 (55.6)	2 (22.2)	2 (22.2)	9 (100.0)	
T descriptor				115	0.06
1–2	28 (48.3)	25 (43.1)	5 (8.6)	58 (100.0)	
3–4	36 (63.2)	13 (22.8)	8 (14.0)	57 (100.0)	
N descriptor				108	0.52
0	10 (45.5)	10 (45.5)	2 (9.1)	22 (100.0)	
1–2–3	47 (54.7)	28 (32.6)	11 (12.8)	86 (100.0)	
M descriptor				128	0.85
0	29 (58.0)	16 (32.0)	5 (10.0)	50 (100.0)	
1	42 (53.8)	26 (33.3)	10 (12.8)	78 (100.0)	
Disease stage				126	0.14
I–II	12 (48.0)	12 (48.0)	1 (4.0)	25 (100.0)	
III–IV	57 (56.4)	30 (29.7)	14 (13.9)	101 (100.0)	
Grading				54	0.62
1	5 (55.6)	4 (44.4)	0 (0)	9 (100.0)	
2	4 (33.3)	6 (50.0)	2 (16.7)	12 (100.0)	
3	15 (45.3)	12 (36.4)	6 (18.2)	33 (100.0)	
Response rate to 1st line therapy				73	0.92
No	28 (62.2)	12 (26.7)	5 (11.1)	45 (100.0)	
Yes	17 (60.7)	7 (25.0)	4 (14.3)	28 (100.0)	

*CNG* copy number gain

### Prognostic analysis

**Fig. 2:**
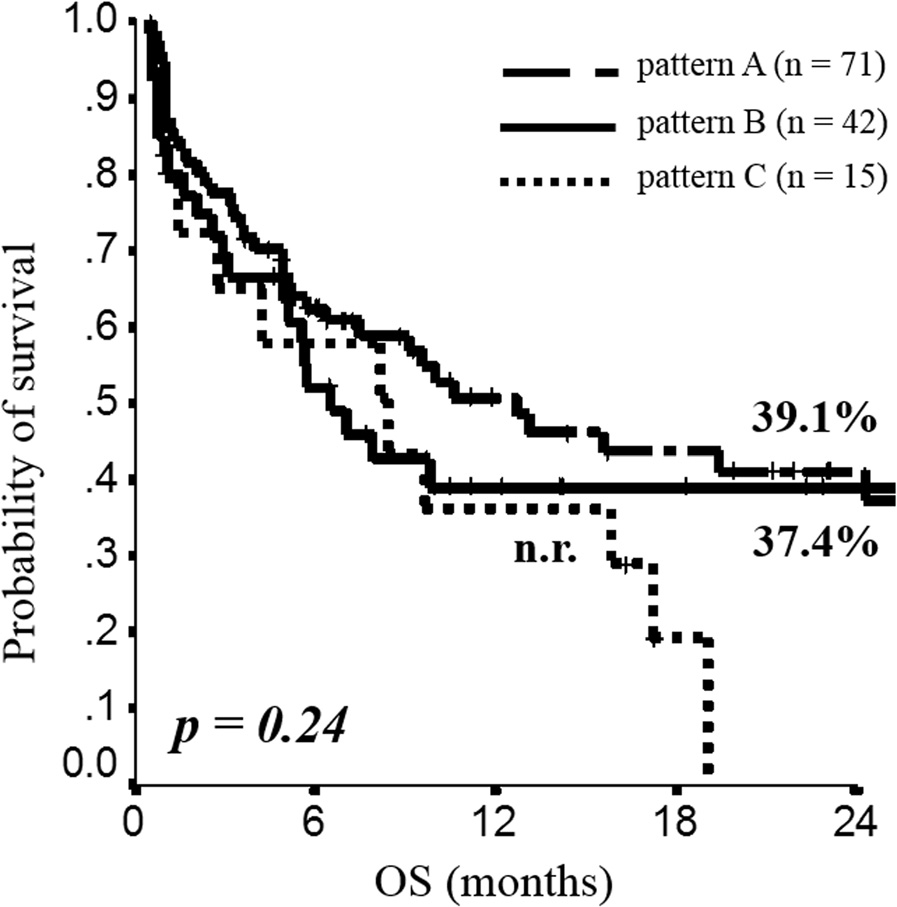
Kaplan Meyer curves for OS in the overall population stratified according to ALK pattern A, B and C

**Fig. 3:**
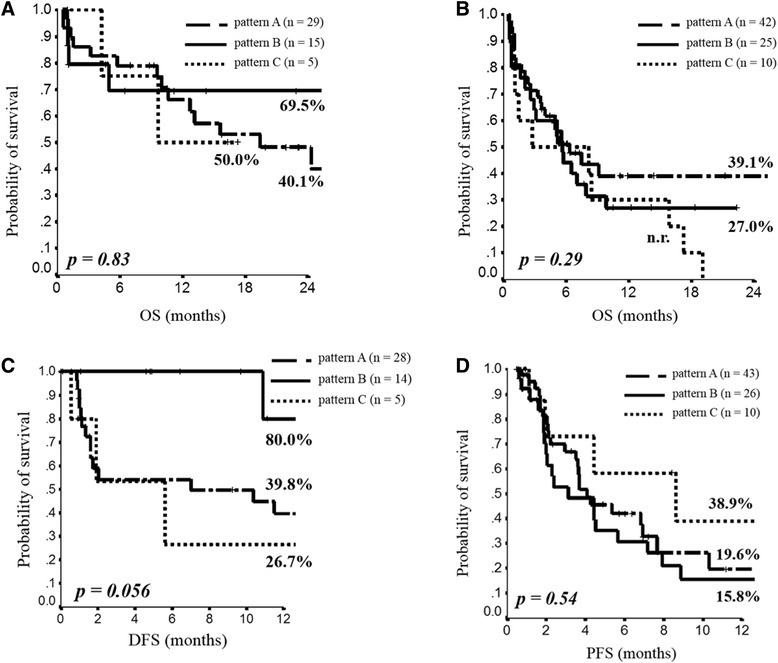
Kaplan Meyer curves stratified according to ALK pattern A, B and C for OS and DFS in resected patients (**a**-**c**) and for OS and PFS in metastatic patients (**b**-**d**)

When all the 128 patients evaluable for the ALK analysis were stratified according to ALK-CNG pattern, no significant difference was observed in terms of survival rate at 2 years (Fig. 2). Among resected patients, although no statistically significant difference was observed, a survival advantage for those patients with ALK-CNG pattern B has been reported (2-year OS 69.5 % versus ≤50 % for pattern A and C) (Fig. 3a). Among metastatic patients, the worst survival was observed for the subgroup of patients with ALK-CNG pattern C (2-year OS 0 % versus 27.0 % and 39.1 % for pattern B and A, respectively) (Fig. 3b). The 1-year DFS among resected patients was increased for those patients with ALK-CNG pattern B (80.0 %) compared to those patients with pattern A (39.8 %) and C (26.7 %) (Fig. 3C). The 1-year PFS among advanced patients did not significantly differ according to the ALK-CNG pattern (15.8 % for pattern A, 19.6 % for pattern B and 38.9 % for pattern C) (Fig. 3D). The univariate analysis confirmed the lack of a statistically significant difference in term of survival according to the ALK-CNG pattern (Table 3). At the univariate analysis, disease stage I-II, tumor size 1-2, negative nodes and surgery were significant predictors for longer DFS in resected patients; whereas performance status 0-1 predicts longer PFS and, together with response to first line chemotherapy and lack of synchronous metastases, longer OS in advanced patients (Table 3).

**Table 3:** Univariate analysis

Variables	Disease free survival			Cancer specific survival			Overall survival		
	HR	95 % CI	*P*	HR	95 % CI	*P*	HR	95 % CI	*P*
Gender [male versus female]	1.213	0.59–2.48	0.596	1.152	0.67–1.96	0.602	1.063	0.70–1.60	0.770
Age [≤66 versus >66 years]	1.709	0.82–3.55	0.151	1.153	0.68–1.94	0.592	1.191	0.79–1.77	0.390
Smoking status [never versus current/former]	1.478	0.60–1.47	0.395	1.782	0.85 - 3.69	0.121	1.332	0.79–2.23	0.278
Comorbidity [no versus yes]	1.395	0.65–2.96	0.388	-	-	-	-	-	-
ALK-CNG^a^ [Disomic/3-7 versus > 7]	1.695	0.49–5.77	0.399	1.498	0.62–3.57	0.363	1.598	0.85–2.98	0.141
Disease stage [I-II versus III]	13.12	4.59–37.4	<0.001	-	-	-	-	-	-
T descriptor [1–2 versus 3–4]	3.920	1.73–8.86	0.001	-	-	-	-	-	-
N descriptor [0 versus 1]	4.259	1.79–10.2	0.001	-	-	-	-	-	-
Grading [1 versus 2 versus 3]	2.128 3.390	0.42–10.6 0.73–15.6	0.357 0.117	-	-	-	-	-	-
Surgery [yes versus no]	8.425	3.65–19.3	<0.001	-	-	-	-	-	-
Performance Status [0 versus 1 versus 2–3]	-	-	-	2.358 4.581	1.25–4.42 1.81–11.5	0.007 0.001	1.647 3.864	1.00–2.70 1.98–7.52	0.049 <0.001
Synchronous Metastases [no versus yes]	-	-	-	1.119	0.15–8.14	0.912	2.335	1.52–3.58	<0.001
Response rate to 1st line [yes versus no]	-	-	-	-	-	-	2.356	1.31–4.23	0.004

*HR* hazard ratios, *CI* confidence intervals, *CNG* copy number gain

^a^The results of the univariate analysis are not significant even categorizing individually the variables [disomic versus 3–7 versus > 7]

## Discussion

Our analysis aimed to investigate the potential correlation between ALK-CNG and clinical features, exploring the prognostic implications of ALK-CNG in a retrospective cohort of 167 NSCLC patients. We decided to exclude from the analysis patients harboring EGFR mutant and ALK rearranged NSCLC in order to avoid additional confounding factors reducing the reliability of the prognostic analysis. In fact, to evaluate the prognostic effect of ALK and EGFR alterations, patients should not be treated with the selective inhibitors commonly used in clinical practice, because the benefit deriving from these agents might mask the prognostic value of the biomarker in-study. Overall, ALK-CNG was observed in the 34.1 % of cases. The 25.1 % of the samples showed ALK fluorescent signal ranging from 3 to 7 and, for all of them, the ALK-CNG was due to polyploidy (ALK/CEPs ratio < 2), whereas only 9 % of tumors showed ALK fluorescent signal > 7. Therefore, similarly to what demonstrated by our group in the study of the 3q chromosomal amplification in squamous cell lung carcinoma [29], ALK-CNG seems mostly related to polyploidy (whole DNA reduplication) rather than locus specific gene amplification.

As reported in other retrospective studies [24], our results suggest a correlation between ALK-CNG and early stage disease (the majority of T1 tumors clusterize in pattern B, none in pattern C). Moreover, smokers and former smokers showed an increased probability to harbor an ALK-CNG compared to never smokers, which are in the 80 % of cases identified in pattern A. These findings support the hypothesis that ALK-CNG might represent a marker of chromosomal instability appearing early in NSCLC carcinogenesis and potentially triggered by cigarette smoking.

Although no definitive conclusions can be drawn from the results of our prognostic analysis, some interesting hypotheses emerged regarding the intrinsic biological features of the three ALK-CNG patterns, potentially reflecting different levels of chromosomal instability. In fact, while for ALK-CNG pattern B a greater 1-year DFS (80.0 %) and 2-year OS (69.5 %) clearly emerged in resected tumors, conversely, for pattern C, the DFS rate was very low (26.7 %) and in the metastatic setting none of these patients survives at 2 year time-point. Regarding the PFS, the greater 1-year PFS observed in patients with ALK-CNG pattern C (38.9 % versus 19.6 % for pattern A and 15.8 % for pattern B) might reflect the higher chemo-sensitivity of these tumors featured by a strong chromosomal instability. Although in our work we calculate a 2-year survival rate, future studies should be performed with long-term survival outcomes in order to reliably evaluate the prognostic role of a biomarker for clinical setting.

Similarly to our analysis, some other studies evaluating the incidence and potential prognostic implications of ALK gene aberrations have been performed in lung cancer with debatable results. In the context of a retrospective analysis of 107 NSCLC cases, ALK amplification (>5 copies of ALK per cell in 10 % of analyzed cells) and ALK-CNG (mean copy number of 3–5 in 10 % of cells) were identified in the 10 % and 63 % of NSCLC patients, respectively. Although no significant correlation between ALK and clinico-pathological features or prognosis emerged, a significant association between ALK amplification and early stage has been reported, supporting the hypothesis that ALK amplification might represent an early genetic event in NSCLC and potential marker of genomic instability [30]. In a retrospective analysis of 20 pulmonary sarcomatoid carcinomas (PSC), the frequency of ALK-CNG was significantly higher compared to NSCLC with adenocarcinoma histology (22 % versus 0.02 %) with a mean copy number gain of 7 and a significant association with chromosome 7 (EGFR) and 17 (HER2) polysomy. This finding suggests the implication of ALK-CNG as an oncogenic event in PSC, potentially correlated with epithelial-mesenchymal transition and sarcomatoid differentiation [31]. Future confirmation of the oncogenic role of ALK-CNG could support the design of selective studies exploring the potential activity of the ALK inhibition in PSC. Another study conducted in patients with NSCLC and brain metastases reported ALK-CNG in the 11 % of cases with an interesting increased ALK-CNG in brain metastases compared to primary tumors, supporting the rationale of ALK-CNG as a genetic aberration connected to aggressiveness and metastatic behavior [32]. Moreover, in a large retrospective analysis of 1500 NSCLC patients, ALK-CNG (mean native copy number ranged from 2 to 7) was reported in the 80 % of cases and was significantly more common in ALK non-rearranged tumors compared to the translocated ones (62 % versus 19 %). Furthermore, as observed in our analysis, ALK-CNG was mostly related to polysomy, whereas focal amplification was a rare event (<2 % in ALK non-rearranged tumors) [33]. Regarding ALK inhibitors, ALK-CNG, besides representing a marker of insensitivity to crizotinib, was reported to be a mechanism of resistance to crizotinib in ALK-translocated NSCLC both in vitro [34] and in patients progressing during crizotinib treatment [35]. To summarize, our analysis supports the fact that, although ALK pattern B seems to appear early in the tumorigenesis of NSCLC and might mirror an early genomic instability, an high ALK gene copy number (>7, pattern C) related to polyploidy, could be considered as a cut off to discriminate genomically unstable and smoking-related NSCLC featured by an aggressive biological behavior, frequently in advanced stage and with a poor awaited prognostic outcome.

## Conclusion

Although limited by the retrospective nature, the results of our analysis are able to generate interesting hypotheses regarding the biological behavior and the potential therapeutic implication of ALK genetic aberrations, ALK-CNG in particular. As observed in other studies, our analysis confirms that a high ALK gene copy number gain is not a driver genetic event in lung cancer tumorigenesis but it might represent a marker of chromosome instability, correlated with an aggressive metastatic behavior. In this regard, early pathogenic events probably induce different ALK aberrant NSCLC. The ALK non-translocated tumors are frequently associated with chromosomal instability and ALK-CNG, whereas the ALK-translocated NSCLC presents a low native ALK copy number and the increase in ALK-CNG emerges as a mechanism of resistance to crizotinib treatment. Unfortunately, conversely to ALK and ROS1 translocations that are widely recognized oncogenic drivers in a small subset of NSCLC able to predict sensitivity to specific inhibitors, ALK-CNG represents a candidate mechanism of resistance to this target therapy. Therefore, it is mandatory to clarify the role and mechanism of this genetic aberration in order to identify and validate effective therapeutic approaches in this subpopulation of lung cancer patients harboring ALK-CNG. In this context, our analysis is conceived and designed as a further step towards a more biologically rationale approach to treat NSCLC patients. The implications for prognosis should be prospectively investigated and validated in larger patients’ series.

## Abbreviations

CEP: Centromeric alpha-satellite specific for chromosome
CNG: Copy number gain
CR: Complete response
DFS: Disease free survival
ERMS: Embryonal RMS
FISH: Fluorescence in situ hybridization
IBC: Inflammatory breast cancer
NBL: Neuroblastoma
NSCLC: Non-small cell lung cancer
OS: Overall survival
PD: Progressive disease
PFS: Progression free survival
PR: Partial response
PSC: Pulmonary sarcomatoid carcinomas
RFS: Recurrence free survival
RMS: Rhabdomyosarcoma
SD: Stable disease
